# Supplementary material for: Monoclonal Human Antibodies That Recognise the Exposed N and C Terminal Regions of the Often-Overlooked SARS-CoV-2 ORF3a Transmembrane Protein
Source: Viruses. 2021 Nov 2;13(11):2201. doi: 10.3390/v13112201 (PMC8624585; doi:10.3390/v13112201)
Supplement: Supplementary file 1 [file viruses-13-02201-s001.zip › viruses-1414897-supplementary.pdf]

A

N3aB02 scAb

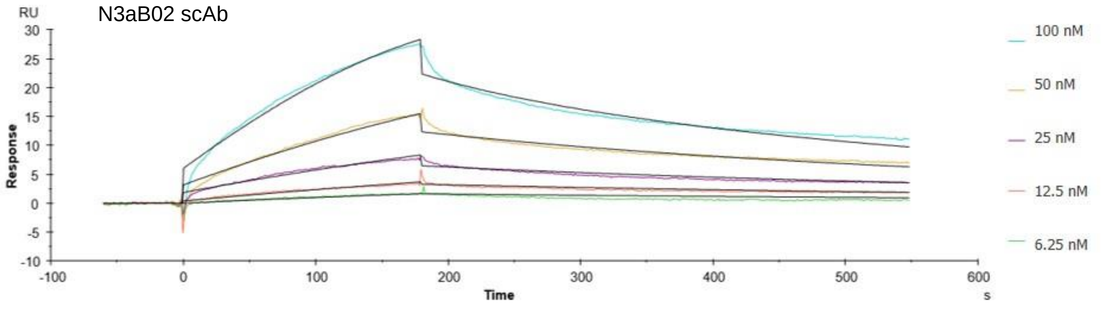

B

3aCA03 scAb

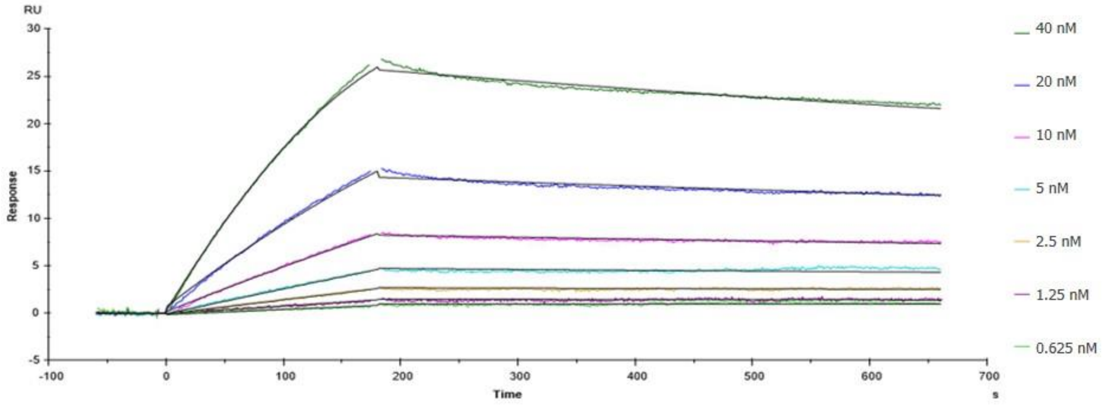

Figure S1. SPR sensorgrams of A) N3aB02 B) 3aCA03 scAbs multi cycle binding kinetics with 1:1 Langmuir model fit as determined by SPR. Coloured lines represent experimental data at various concentrations and black lines represent fitting curves.

A

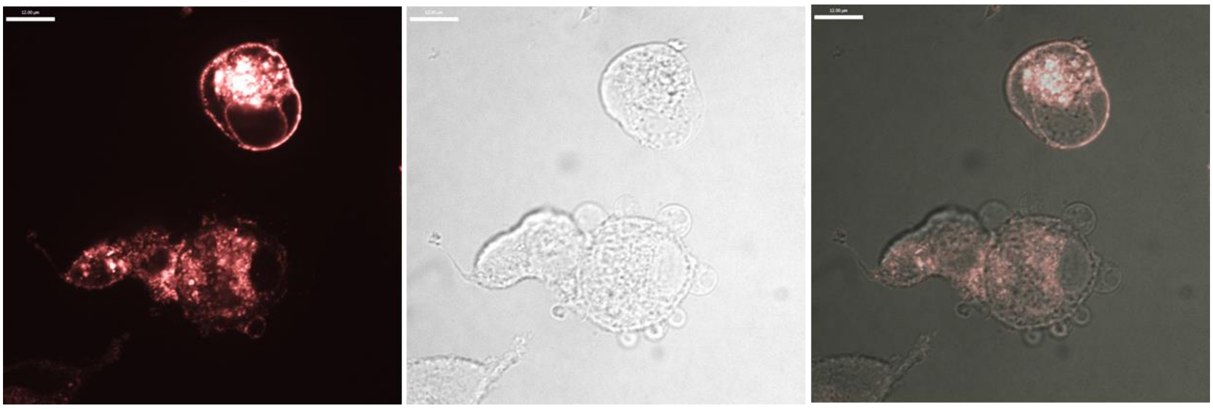

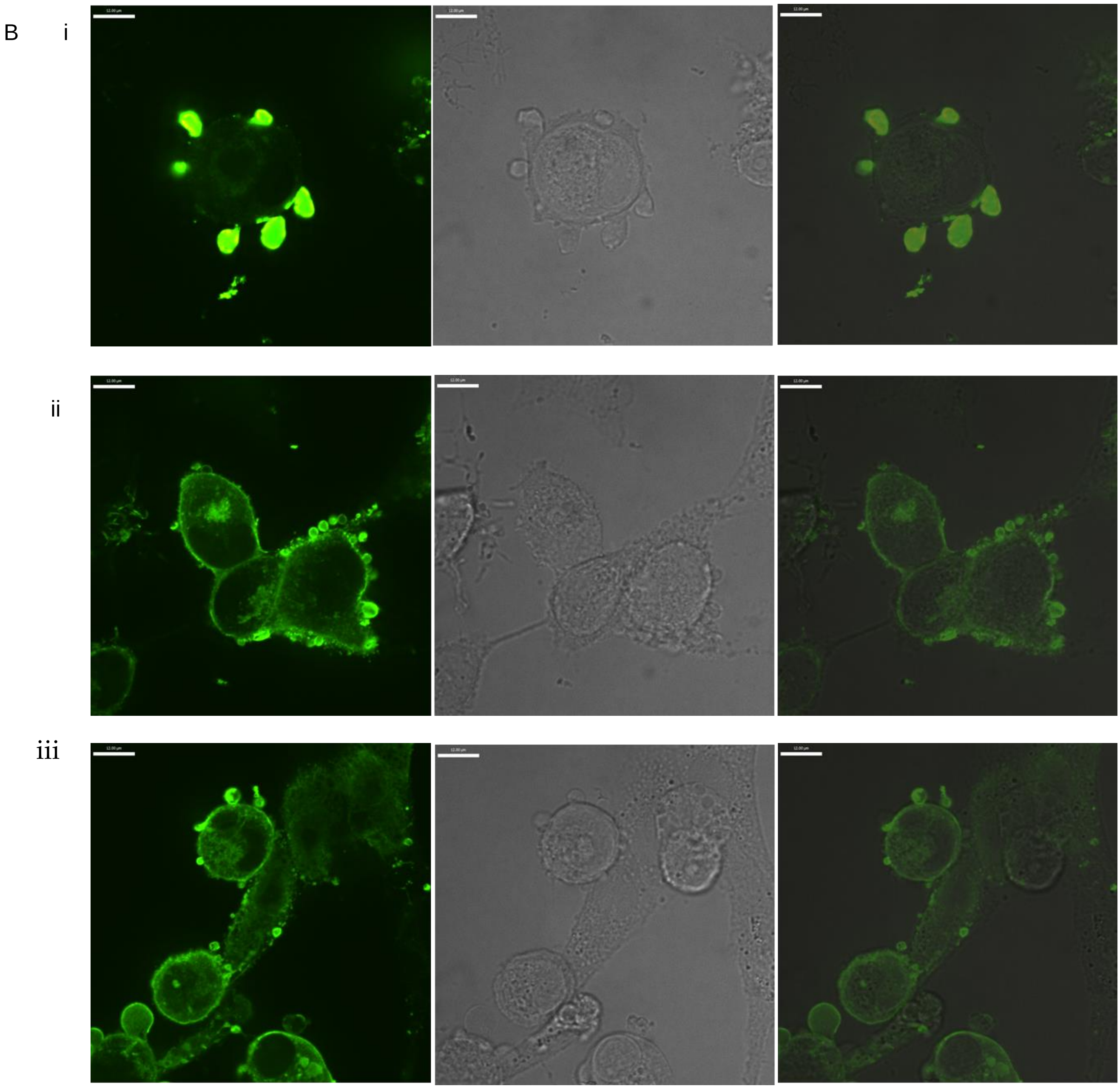

Figure S2. Antibodies staining of swollen 'blebbing' SARS-CoV-2 infected cells. (A) Anti-RBD mAb (40592-R001, Sinobiological) staining in permeabilised cells (B) 3aCA03 scAb staining in (i) non-permeabilised, (ii & iii) permeabilised cells, scale bar = 12μm).
